# Short-form video exposure characteristics and respiratory symptom severity among patients with chronic obstructive pulmonary disease: a cross-sectional study

**DOI:** 10.3389/fdgth.2026.1955608

**Published:** 2026-09-25

**Authors:** Haihong Zhang, Wenxue Zhang, Li Tang

**Affiliations:** 1 School of Nursing, Ningxia Medical University, Ningxia Hui Autonomous Region, Yinchuan, China; 2 School of Medical Information and Engineering, Ningxia Medical University, Yinchuan, Ningxia, China

**Keywords:** breathlessness cough and sputum scale, chronic obstructive pulmonary disease, respiratory symptoms, self-management, short-form video, social media

## Abstract

**Background:**

Short-form video platforms have become a dominant health information source for COPD patients, yet the relationship between content characteristics of exposure and respiratory symptom severity remains unexplored. Given China's 1.02 billion short-form video users with daily viewing exceeding 150 min, understanding differential health impacts of various content types is critical, particularly for older adults vulnerable to algorithmic misinformation.

**Objective:**

This study investigated associations between multidimensional short-form video exposure characteristics—content type (health preservation, illness prevention, daily exercise), viewing duration, and user acceptance—and respiratory symptom severity measured by the BCSS among outpatients with COPD.

**Methods:**

A multi-center cross-sectional study enrolled 207 GOLD-confirmed COPD patients (mean age 63.0 ± 12.9 years; 55.1% male) from January to July 2025. Short-form video exposure was assessed using a validated self-designed questionnaire (Cronbach‘s *α*=0.78, CVI=0.85). Respiratory symptoms were measured using the BCSS. Analyses included t-tests, ANOVA, and hierarchical multiple linear regression, with established clinical confounders forced into multivariable models per STROBE guidelines. Multicollinearity and model assumptions were verified.

**Results:**

Mean BCSS was 7.86 ± 2.24, indicating moderate-to-severe symptom burden. Health preservation content exposure was independently associated with higher BCSS (B = 1.163, *β*=0.227, *P* = 0.003), while daily exercise content showed an inverse association (B = −0.876, *β*=−0.196, *P* = 0.007). Daily viewing duration was not independently associated with BCSS (B = 0.059, *P* = 0.377). Acceptance showed a significant non-linear pattern suggestive of an inverted U-shaped relationship with BCSS (P for non-linearity=0.025), warranting cautious interpretation. Content and acceptance variables improved model fit beyond clinical covariates and duration (*Δ*R^2^ = 0.108, *P* = 0.001), whereas duration alone did not (*Δ*R^2^ = 0.009, *P* = 0.155). The final model explained 22.0% of BCSS variance.

**Conclusion:**

Content type—not duration alone—was significantly associated with respiratory symptom severity in COPD patients. Health preservation content (often non-evidence-based) was linked to worse symptoms, while daily exercise content showed protective associations. The non-linear acceptance-symptom relationship suggests that the association between content acceptance and symptom severity is complex and not simply monotonic; the underlying mechanisms merit further investigation. These findings support content-aware digital health literacy interventions and integration of social media exposure assessment into nursing-led COPD management.

## 1 Introduction

Chronic obstructive pulmonary disease (COPD) remains one of the most pressing global public health challenges, ranking as the third leading cause of death worldwide and contributing substantially to morbidity, disability, and healthcare expenditure (1, 2). The Global Initiative for Chronic Obstructive Lung Disease (GOLD) 2025 report reaffirms that COPD is characterized by persistent respiratory symptoms—principally dyspnea, chronic cough, and sputum production—that progressively limit daily activities and reduce quality of life (1). In China, the prevalence of COPD among adults aged 40 years and older is 13.6%, representing the predominant age group affected by the disease and contributing to the overall burden of approximately 99.9 million patients (3). Despite advances in pharmacological treatment and pulmonary rehabilitation, symptom burden remains inadequately controlled in a substantial proportion of patients, highlighting the need to identify modifiable determinants of symptom severity beyond traditional clinical factors.

The rapid proliferation of short-form video platforms—exemplified by Douyin (the Chinese counterpart of TikTok), Kuaishou, and Video Accounts on WeChat—has fundamentally transformed the landscape of health information consumption in China. As of 2025, China has over 1.02 billion short-form video users, with a per capita daily viewing time exceeding 150 min (4). For older adults with chronic diseases such as COPD, these platforms serve not only as entertainment but also as a primary source of health-related information, including disease management guidance, medication advice, dietary recommendations, and exercise tutorials. However, the quality and scientific accuracy of health information disseminated through short-form video platforms vary widely, with a substantial proportion of content lacking evidence-based foundations (4–6). Studies have documented the prevalence of health misinformation on social media platforms, raising concerns about its potential impact on health behaviors and outcomes in vulnerable populations (5–7).

A growing body of research has examined the health correlates of short-form video use, yet the existing evidence base has notable limitations. First, the predominant focus has been on mental health outcomes—including depression, anxiety, sleep disturbance, and addictive behaviors—primarily among adolescents and young adults (8–12); however, a small but growing body of work has begun to examine short-form video exposure in other chronic disease populations, reporting associations with disease-related outcomes (13–15), and to our knowledge no study has yet examined these associations specifically in relation to respiratory symptom severity among patients with COPD. Second, existing studies have overwhelmingly conceptualized exposure in terms of viewing duration, treating short-form video as a homogeneous exposure medium and neglecting the potentially differential effects of specific content types (11, 16). This duration-centric approach fails to capture the heterogeneity of content encountered on algorithm-driven platforms, where a user watching 2 h of evidence-based pulmonary rehabilitation content may have vastly different health outcomes from one watching 2 h of health preservation content (which, based on the existing literature, often contains non-evidence-based claims). Third, the role of user acceptance—the degree to which viewers actively accept and internalize the content to which they are exposed—has not been examined in relation to health outcomes, despite theoretical frameworks suggesting that acceptance is a critical mediator of media effects (17).

The Elaboration Likelihood Model (ELM) of persuasion (18) provides a useful theoretical framework for understanding how short-form video content may influence health outcomes. According to the ELM, the persuasive impact of media messages depends on the extent to which individuals engage in central (thoughtful, analytical) versus peripheral (heuristic, superficial) processing. For patients with COPD exposed to health-related short-form video content, those with higher eHealth literacy may be more likely to engage in central processing, critically evaluating the credibility and scientific basis of health claims, whereas those with lower eHealth literacy may rely on peripheral cues (e.g., visual appeal, emotional tone, number of likes), rendering them more susceptible to potentially harmful non-evidence-based content (19–22). We emphasize that ELM is used in this study as an interpretive framework to aid understanding of the observed associations, rather than as a construct that was directly operationalized or measured; in particular, eHealth literacy, information-processing pathways, and credibility evaluation were not directly measured in this study.

Building on this theoretical framework and the identified gaps in the literature, this study aimed to investigate the associations between multidimensional short-form video exposure characteristics—content type, daily viewing duration, and user acceptance—and respiratory symptom severity measured by the BCSS among patients with COPD. We hypothesized that: (H1) the content type of short-form video exposure would be more strongly associated with BCSS scores than daily viewing duration; (H2) exposure to health preservation content which, based on the existing literature on Chinese social media, often contains non-evidence-based claims would be associated with higher (worse) BCSS scores, while exposure to daily exercise content would be associated with lower (better) BCSS scores; and (H3) acceptance of screen-exposed content would show a significant association with BCSS scores, with the direction and shape of this relationship explored through non-linear modeling. By examining these multidimensional exposure characteristics, this study seeks to advance the field beyond the prevailing duration-centric paradigm and inform the development of content-aware digital health literacy interventions for patients with COPD.

## 2 Methods

### 2.1 Study design and setting

This multi-center cross-sectional study was conducted between January and July 2025 at respiratory outpatient clinics, pulmonary function laboratories, and pulmonary rehabilitation departments of several hospitals in Yinchuan, China. The study was designed and reported in accordance with the Strengthening the Reporting of Observational Studies in Epidemiology (STROBE) guidelines (23). Ethical approval was obtained from the Ethics Committee of Ningxia Medical University (approval number: 2024-N047).

### 2.2 Participants

Eligible participants were community-dwelling adults aged 40 years and older with a confirmed diagnosis of COPD according to the GOLD criteria [post-bronchodilator forced expiratory volume in 1 s [FEV₁]/forced vital capacity [FVC] < 0.70] (1). “Community-dwelling” was defined in this study as patients living in the community who attended outpatient care and were not hospitalized at the time of the survey; participants were recruited from respiratory outpatient clinics, pulmonary function laboratories, and pulmonary rehabilitation departments. The age criterion of at least 40 years was selected because COPD prevalence and severity increase markedly after age 40, and because older adults constitute the primary population of short-form video users for health information in China. Additional inclusion criteria were: (a) ability to communicate effectively in Mandarin; (b) ownership of a smartphone with active short-form video application usage (Douyin, Kuaishou, or WeChat Video Accounts); and (c) minimum short-form video viewing history of 3 months. Exclusion criteria were: (a) language or hearing impairment precluding effective communication; (b) a concurrent diagnosis of pneumonia, bronchiectasis, or other major pulmonary diseases (e.g., lung cancer, tuberculosis, interstitial lung disease); and (c) an acute exacerbation of COPD within the preceding 4 weeks. A total of 207 patients were enrolled using convenience sampling.

### 2.3 Sample size

The sample size was calculated *a priori* for hierarchical multiple regression analysis. We performed a formal a-priori power calculation using G*Power 3.1 for multiple linear regression with a two-tailed *α* = 0.05 and a medium effect size (f^2^ = 0.15). The required sample size was 196. In addition to this formal power calculation, we also considered the widely recommended rule of thumb requiring 10–15 observations per predictor variable for stable regression estimates. Anticipating a final model with up to 21 predictors, this heuristic suggested a target of approximately 210 participants. We therefore aimed to recruit 210 participants, while the formal power calculation (*N* = 196) served as the lower bound for adequate power.

### 2.4 Measures

#### 2.4.1 Dependent variable: BCSS

The primary outcome variable was respiratory symptom severity, measured using the Breathlessness, Cough, and Sputum Scale (BCSS) (24). The BCSS is a interviewer-administered, three-item rating scale that assesses the severity of breathlessness, cough, and sputum production on a 5-point Likert scale ranging from 0 (none) to 4 (severe). Total scores range from 0 to 12, with higher scores indicating greater respiratory symptom burden. The BCSS has been validated in COPD populations and shown to predict exacerbation risk (25), the total BCSS score of 5.0 was identified as the optimal cut-off value for detecting acute exacerbation. In this study, the BCSS was administered by trained research nurses during face-to-face interviews.

#### 2.4.2 Independent variables: short-form video exposure questionnaire

A self-designed questionnaire was developed to assess short-form video exposure, based on relevant literature on this topic (26–29) and a qualitative pre-study elicitation in which patients with COPD described the categories of short-form video content they most frequently encountered. The short-form video exposure questionnaire encompassed three core dimensions: (1) daily viewing duration, categorized into five levels (0–1, 2–3, 4–5, 6–7, and ≥8 h); these bands were chosen based on the empirically observed distribution of self-reported daily viewing time, which was highly right-skewed, and to ensure an adequate number of participants in each band. For the open-ended ≥8 h/day category, the midpoint was calculated as the lower bound plus half the width of the preceding interval (i.e., 9 h); midpoint values were used for regression analyses; (2) content types, including sharing of daily exercise, health preservation knowledge, and illness prevention knowledge, each recorded as a binary variable; and (3) acceptance of screen-exposed content, measured on a single-item 5-point ordinal scale (1 = complete rejection to 5 = full acceptance). This single-item measure was used because acceptance was conceptualized as a single, global, unidimensional self-report construct (18). The content categories were validated through an expert-panel procedure in which five experts (in respiratory medicine, nursing, and health communication) rated the relevance of each content-category item on a four-point scale; the resulting item-level content validity index (I-CVI) was 0.85. The expert panel employed a ≥ 50% agreement threshold for item retention. The questionnaire demonstrated acceptable content validity (CVI = 0.85) in a pilot sample of 30 patients. In this pilot sample, the content categorization demonstrated an 82% concordance rate with the tags assigned by content creators/platforms, supporting the validity of the classification. Regarding internal consistency, Cronbach's α  = 0.78 was calculated for the items assessing exposure frequency across the three content types (daily exercise, health preservation, and illness prevention) and daily viewing duration; the single-item acceptance measure was excluded from this calculation because Cronbach's α is not interpretable for single-item constructs. We note that exposure to misinformation was not directly measured in this study, and that the characterization of health preservation content as potentially non-evidence-based is drawn from the published literature rather than from direct measurement of misinformation.https://www.frontiersin.org/journals/digital-health/articles/10.3389/fdgth.2026.1761107/xml

#### 2.4.3 Covariates: demographic and clinical variables

Demographic and clinical data were collected through structured interviews and medical record review, including: gender (male/female), ethnicity (Han/other), residence (urban/rural), marital status (unmarried/married), education level (no formal education/primary education/junior secondary education/post-secondary education), BMI (<18.5, 18.5–23.9, ≥24.0 kg/m^2^ based on Chinese cut-off points) (30)), disease duration, comorbidity burden measured by the Functional Comorbidity Index (FCI) (31), exacerbation phenotype (0–1 and ≥2 acute exacerbations in the preceding 12 months), smoking status (never/ever/current), and secondhand smoke (SHS) exposure (yes/no).

### 2.5 Statistical analysis

Continuous variables were expressed as mean ± standard deviation (SD) and categorical variables as frequencies (percentages). Between-group differences in BCSS scores were assessed using independent-samples t-tests for two-group comparisons and one-way analysis of variance (ANOVA) with least significant difference (LSD) *post hoc* comparisons for multi-group comparisons. Normality of distributions was verified through the Shapiro–Wilk test and visual inspection of Q-Q plots. Homogeneity of variance was confirmed using Levene's test.

Hierarchical multiple linear regression was employed to examine the independent associations between short-form video exposure variables and BCSS scores while controlling for potential confounders. Based on COPD guideline recommendations (1) and methodological best practices (32, 33), the following clinical variables were forced into all multivariable models regardless of their univariate *P*-values, as they are established determinants of COPD symptom severity: smoking status (never/ever/current, with never as reference), secondhand smoke exposure (yes/no, with no as reference), disease duration (continuous, in years), and comorbidity burden (FCI score, continuous). Additional variables with *P* < 0.30 in univariate analyses (gender, BMI category, education level, exacerbation phenotype) were also included. This approach ensures that known clinical confounders are controlled even when not statistically significant in the present sample, reducing the risk of residual confounding.

Variable coding for the multivariable model was as follows: continuous variables (disease duration in years, FCI score, and daily viewing duration in midpoint hours) were entered without transformation; categorical variables were dummy-coded with clinically meaningful reference categories: gender (female = reference), smoking status (never = reference), SHS exposure (no = reference), education level (no formal education = reference), BMI (18.5–23.9 = reference), exacerbation phenotype (0–1 = reference), content type variables (no exposure = reference for each), and acceptance of screen-exposed content (complete rejection = reference). No centering was applied to continuous variables given the absence of interaction terms in the primary models. Multiple comparisons were addressed through the multivariable model itself, which adjusts for all included covariates simultaneously; no additional correction (e.g., Bonferroni) was applied to the multivariable regression results, as the analysis was hypothesis-driven rather than exploratory.

The hierarchical regression comprised three strata: Stratum 1 included demographic and clinical covariates only (gender, BMI, disease duration, smoking status, SHS exposure, education level, exacerbation phenotype, FCI score); Stratum 2 added daily short-form video viewing duration (continuous); Stratum 3 added content type variables (daily exercise, health preservation knowledge, and illness prevention knowledge) and acceptance of screen-exposed content (categorical). The incremental contribution of each stratum was assessed using the change in R^2^ (*Δ*R^2^) and its associated F-change test, calculated as: *Δ*F = [(R^2^_new−R^2^_old)/(k_new−k_old)]/[(1−R^2^_new)/(n−k_new−1)], where k represents the number of predictors and n the sample size.

Multicollinearity was assessed using variance inflation factors (VIFs), with VIF < 5 considered acceptable (34). Model assumptions of normality, homoscedasticity, and independence of residuals were verified through residual analysis and the Durbin-Watson test. Restricted cubic spline (RCS) regression with 4 knots (at the 5th, 35th, 65th, and 95th percentiles) was used to model potential non-linear relationships between continuous exposure variables (daily viewing duration and acceptance score) and BCSS (35). Although acceptance was originally measured on a five-point ordinal scale, we treated it as a continuous variable in the restricted cubic spline regression for the following reasons. First, the ordinal categories reflect an underlying continuum of acceptance intensity, and treating them as continuous allows the data to reveal the shape of the dose-response relationship without imposing a linear assumption. Second, the frequency distribution across the five categories (ranging from 5.8% to 28.5%) provided sufficient information to estimate the spline parameters. Third, this approach has been used in prior methodological work on dose-response analysis with ordinal exposures (32, 36). Nonetheless, we acknowledge the ordinal nature of the variable and performed sensitivity analyses treating acceptance as an ordered categorical variable to confirm the robustness of the findings (see Results). The knot locations for the restricted cubic spline were placed at the 5th, 35th, 65th, and 95th percentiles of the acceptance score distribution, following the default recommendation in the rms package (32). The overall association was tested using the Wald test for the spline terms, and non-linearity was specifically tested by comparing the model with only the linear term to the model including both linear and non-linear spline terms (P for non-linearity).

Data completeness was verified prior to analysis; no missing data were present in the analytic dataset (all 207 participants provided complete responses for all study variables), and therefore no imputation or listwise deletion was necessary. All statistical analyses were performed using IBM SPSS Statistics version 26.0 (IBM Corp., Armonk, NY, USA) and R version 4.3.0 (R Foundation for Statistical Computing, Vienna, Austria) with the rms package for RCS analysis. Two-tailed *P* < 0.05 was considered statistically significant.

## 3 Results

### 3.1 Participant characteristics

A total of 277 patients with COPD were screened for eligibility across the participating hospitals. Seventy patients were excluded according to the predefined criteria (23 due to language or hearing impairment, 28 due to concurrent diseases such as pneumonia and bronchiectasis, and 19 due to an acute exacerbation within the preceding 4 weeks), yielding 207 eligible patients (74.7% of those screened), all of whom consented and completed the survey. The participant flow is shown in Figure 1. The mean age was 63.00 ± 12.90 years, with 55.07% male and 89.37% of Han ethnicity. The mean BMI was 23.42 ± 3.86 kg/m^2^, with 9.66% being underweight (BMI < 18.5). The mean disease duration was 7.04 ± 6.59 years, and 64.25% had experienced ≥2 acute exacerbations in the preceding 12 months. The mean FCI score was 2.54 ± 1.31. Regarding smoking status, 69.08% were never-smokers, 19.81% were ever-smokers, and 11.11% were current smokers. Secondhand smoke exposure was reported by 27.05% of participants (Table 1). The mean BCSS score was 7.86 ± 2.24, indicating moderate-to-severe respiratory symptom burden (Table 2). The distribution of acceptance of screen-exposed content scores was as follows: complete rejection (*n* = 56, 27.1%), minor acceptance (*n* = 59, 28.5%), moderate acceptance (*n* = 56, 27.1%), major acceptance (*n* = 24, 11.6%), and full acceptance (*n* = 12, 5.8%). The proportion of participants in each category ranged from 5.8% to 28.5%, indicating sufficient variation for the non-linear modeling (Table 3).

**Figure 1:**
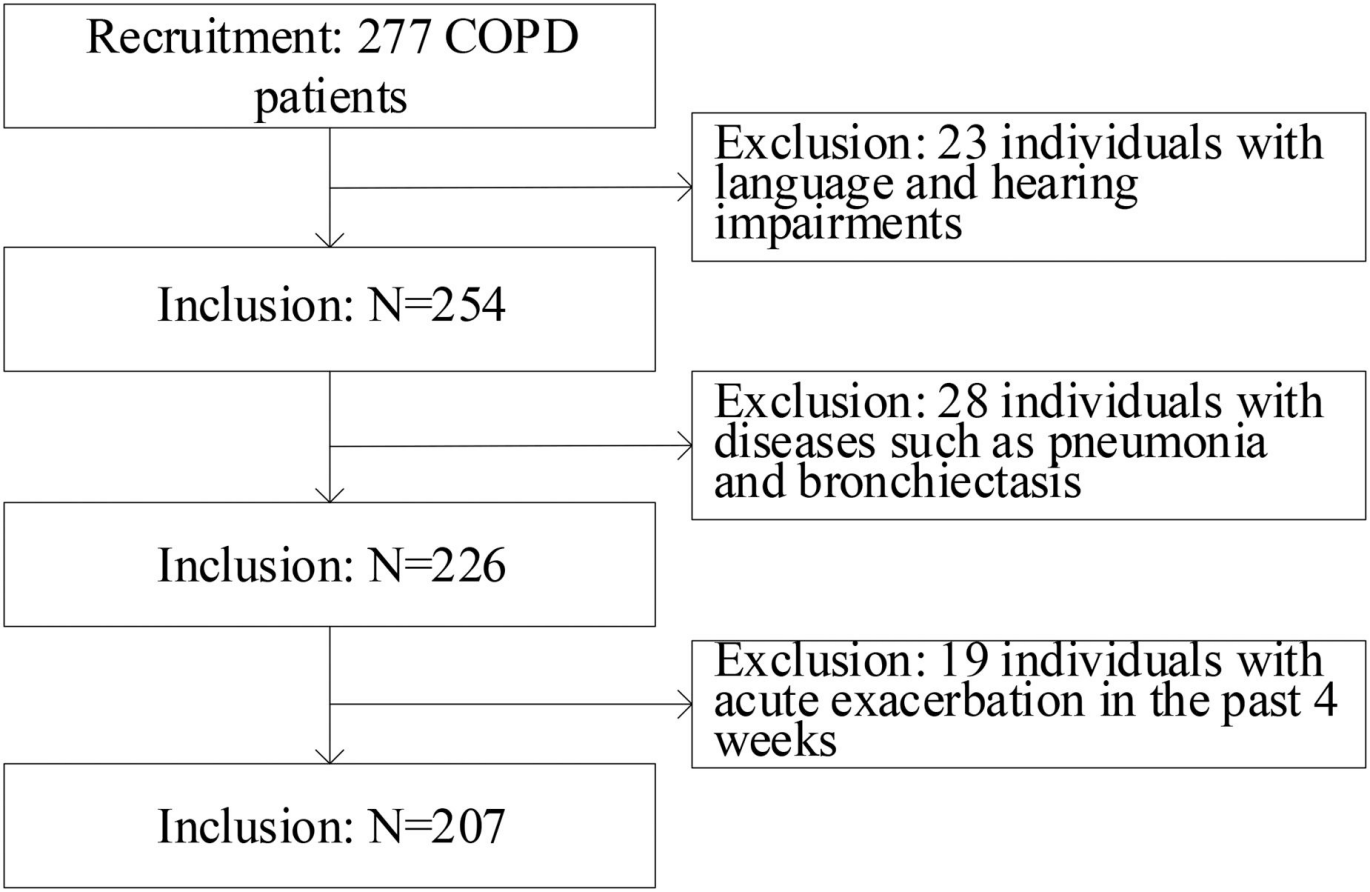
Flow of recruiting COPD patients.

**Table 1:** Baseline characteristics and of participants and univariate analysis associations with BCSS (*n*=207).

Variables	Frequencies (n)	Percentage (%)	BCSS(M ± SD)	*F/t*	*p*
Gender				1.552	0.123
Male(*n*=114)	114	55.07	8.08 ± 1.94		
Female(*n*=93)	93	44.93	7.58 ± 2.56		
Ethnicity				0.784	0.434
Han(*n*=185)	185	89.37	7.90 ± 2.21		
Others(*n*=22)	22	10.63	7.50 ± 2.58		
Residence				1.029	0.305
Urban(*n*=65)	65	31.40	8.09 ± 2.20		
Rural(*n*=142)	142	68.60	7.75 ± 2.26		
Passive smoking				0.549	0.584
No	151	72.95	7.91 ± 2.25		
Yes	56	27.05	7.71 ± 2.25		
Smoking status				0.094	0.910
Never	143	69.08	7.84 ± 2.38		
Ever	41	19.81	7.80 ± 2.09		
Current	23	11.11	8.04 ± 1.58		
BMI				3.441	0.034*
＜18.5	20	9.66	9.00 ± 2.25		
18.5∼23.9	105	50.72	7.59 ± 2.27		
≥24.0	82	39.61	7.91 ± 2.14		
Exacerbation phenotype				−1.772	0.078
0–1	74	35.75	7.49 ± 1.92		
≥2	133	64.25	8.06 ± 2.39		
Marital status				0.459	0.647
Unmarried	69	33.33	7.96 ± 2.32		
Married	138	66.67	7.80 ± 2.21		
Education level				2.299	0.079
No formal	106	51.21	7.55 ± 2.37		
Primary	40	19.32	8.20 ± 1.95		
Junior	36	17.39	8.56 ± 1.89		
Post	25	12.08	7.60 ± 2.42		

* *p*<0.05 **p<0.01.

**Table 2:** Scores of continuous variables and their relationship with BCSS.

Variables	Mean ± SD	Correlation with BCSS (r)
BCSS	7.86 ± 2.24	
Disease duration	7.04 ± 6.59	0.032, *P* = 0.645
FCI score	2.54 ± 1.31	−0.021, *P* = 0.765

**Table 3:** Univariate analysis of short-form exposure characteristics and their impact on BCSS (*n*=207).

Variables	Frequencies (percentage)	BCSS(M ± SD)	*t*/F	*p*
Acceptance of screen-exposed content			3.268	0.013*
Complete rejection	56 (27.05)	7.41 ± 2.20		
Minor acceptance	59 (28.50)	8.25 ± 2.07		
Moderate acceptance	56 (27.05)	8.41 ± 2.33		
Major acceptance	24 (11.59)	7.13 ± 1.87		
Full acceptance	12 (5.80)	6.83 ± 2.72		
Sharing of daily exercise			1.568	0.119
No	105 (50.72)	8.10 ± 2.20		
Yes	102 (49.28)	7.61 ± 2.27		
Sharing of health preservation knowledge			−4.196	＜0.001**
No	154 (74.40)	7.53 ± 2.30		
Yes	53 (25.60)	8.81 ± 1.78		
Sharing of illness prevention knowledge			−2.096	0.037*
No	164 (79.23)	7.69 ± 2.23		
Yes	43 (20.77)	8.49 ± 2.23		
Daily duration of short-form video exposure/hours			2.811	0.027*
0–1	110 (53.14)	7.55 ± 2.17		
2–3	39 (18.84)	8.15 ± 2.76		
4–5	24 (11.59)	7.75 ± 2.17		
6–7	22 (10.63)	9.18 ± 1.22		
≥8	12 (5.80)	7.42 ± 1.88		

For two-group comparisons (daily exercise, health preservation, illness prevention), independent-samples t-test was used, with the test statistic reported as t. For multi-group comparisons (acceptance, daily duration), one-way ANOVA was used, with the test statistic reported as F. The column header “Test statistic” reflects this distinction.

*p < 0.05

**p < 0.001

### 3.2 Univariate associations with BCSS scores

In univariate analyses (Tables 1–3), BMI category (F = 3.441, *P* = 0.034), exacerbation phenotype (t = −1.772, *P* = 0.078, marginal), education level (F = 2.299, *P* = 0.079, marginal), daily viewing duration (F = 2.811, *P* = 0.027), exposure to illness prevention knowledge videos (t = −2.096, *P* = 0.037), exposure to health preservation videos (t = −4.196, *P* < 0.001), and acceptance of screen-exposed content (F = 3.268, *P* = 0.013) were significantly or marginally associated with BCSS scores. Notably, the acceptance-BCSS relationship exhibited a non-monotonic pattern: BCSS scores increased from complete rejection (7.41 ± 2.20) through minor acceptance (8.25 ± 2.07) and moderate acceptance (8.41 ± 2.33), then decreased at major acceptance (7.13 ± 1.87) and full acceptance (6.83 ± 2.72), suggesting an inverted U-shaped relationship.

Among the short-form video content types, health preservation knowledge exposure was associated with the largest difference in BCSS scores (8.81 ± 1.78 vs. 7.53 ± 2.30; *P* < 0.001), followed by illness prevention knowledge (8.49 ± 2.23 vs. 7.69 ± 2.23; *P* = 0.037). Daily exercise sharing did not reach statistical significance in univariate analysis (8.10 ± 2.20 vs. 7.61 ± 2.27; *P* = 0.119), though its association became significant after multivariable adjustment (see below), consistent with a suppression effect (37).

### 3.3 Multivariable regression analysis

Table 4 presents the results of the multivariable linear regression analysis with all forced and selected covariates. The overall model was statistically significant [F (20, 186) = 2.627, *P* < 0.001] and explained 22.0% of the variance in BCSS scores (R^2^ = 0.220, adjusted R^2^ = 0.136). The Durbin-Watson statistic was 1.749, indicating no significant autocorrelation, and all VIFs were below 2.3, confirming the absence of problematic multicollinearity.

**Table 4:** Multivariable analysis of the association between short-form video exposure and respiratory symptoms (*n*=207).

Variables	Unstandardized		Standardized	*t*	*p* Value	Collinearity Diagnostic	
	*B*	Std. Error	*Beta*			VIF	Tolerance
Constant	6.489	1.003	-	6.472	＜0.001**	-	-
Gender							
Female (Ref.)							
Male	0.001	0.350	<0.001	0.003	0.997	1.443	0.693
Disease duration	0.009	0.024	0.026	0.370	0.712	1.184	0.845
Smoking status							
Never smoking (Ref.)							
Ever smoking	−0.434	0.440	−0.077	−0.987	0.325	1.464	0.683
Current smoking	−0.191	0.569	−0.027	−0.335	0.738	1.523	0.657
Passive smoking							
No (Ref.)							
Yes	0.170	0.393	0.034	0.432	0.666	1.453	0.688
FCI score	0.010	0.151	0.006	0.069	0.945	1.855	0.539
Education level							
No formal (Ref.)							
Primary	0.508	0.470	0.090	1.081	0.281	1.636	0.611
Junior	0.847	0.517	0.143	1.639	0.103	1.826	0.548
Post	−0.214	0.585	−0.031	−0.366	0.715	1.727	0.579
BMI							
18.5–23.9 (Ref.)							
＜18.5	1.150	0.561	0.152	2.049	0.042*	1.307	0.765
≥24.0	0.178	0.322	0.039	0.554	0.580	1.180	0.847
Exacerbation phenotype							
0∼1 (Ref.)							
≥2	0.823	0.337	0.176	2.447	0.015*	1.238	0.808
Daily duration of short-form video exposure/hours	0.059	0.066	0.084	0.886	0.377	2.145	0.466
Sharing of daily exercise							
No (Ref.)							
Yes	−0.876	0.324	−0.196	−2.704	0.007**	1.247	0.802
Sharing of health preservation knowledge							
No (Ref.)							
Yes	1.163	0.386	0.227	3.009	0.003**	1.353	0.739
Sharing of illness prevention knowledge							
No (Ref.)							
Yes	0.562	0.430	0.102	1.307	0.193	1.450	0.690
Acceptance of screen-exposed content							
Complete rejection (Ref.)							
Minor acceptance	0.556	0.419	0.112	1.325	0.187	1.706	0.586
Moderate acceptance	0.183	0.485	0.036	0.377	0.707	2.213	0.452
Major acceptance	−0.657	0.563	−0.094	−1.168	0.244	1.545	0.647
Full acceptance	−1.036	0.710	−0.108	−1.460	0.146	1.309	0.764
R-squared	0.220						
Adjust R-squared	0.136						
*F* Test	*F* (20,186)=2.627,*p*=<0.001						
D-W	1.749						

Dependent Variable = BCSS. *p<0.05 **p<0.01.

Among the short-form video exposure variables, exposure to health preservation content was positively and independently associated with BCSS scores (B = 1.163, SE = 0.386, *β* = 0.227, t = 3.009, *P* = 0.003), indicating that patients exposed to such content had significantly higher respiratory symptom severity after adjusting for all clinical and demographic covariates. Conversely, exposure to daily exercise content was inversely associated with BCSS scores (B = −0.876, SE = 0.324, *β* = −0.196, t = −2.704, *P* = 0.007), demonstrating a protective association. Exposure to illness prevention knowledge content showed a positive but non-significant association (B = 0.562, *P* = 0.193). Daily viewing duration was not independently associated with BCSS (B = 0.059, *β* = 0.084, *P* = 0.377).

Regarding acceptance of screen-exposed content, while none of the individual acceptance categories reached statistical significance compared with the reference (complete rejection), the pattern of coefficients was consistent with the inverted U-shaped relationship observed in univariate analysis: minor acceptance (B = 0.556, *P* = 0.187) and moderate acceptance (B = 0.183, *P* = 0.707) showed positive coefficients, while major acceptance (B = −0.657, *P* = 0.244) and full acceptance (B = −1.036, *P* = 0.146) showed negative coefficients. This pattern was further explored through RCS analysis as an exploratory finding (see Section 4.5).

Among the clinical covariates, exacerbation phenotype (≥2 vs. 0–1; B = 0.823, *β* = 0.176, *P* = 0.015) and being underweight (BMI < 18.5 vs. 18.5–23.9; B = 1.150, *P* = 0.042) were the only clinical variables independently associated with BCSS. The forced clinical confounders—smoking status (ever: B = −0.434, *P* = 0.325; current: B = −0.191, *P* = 0.738), secondhand smoke exposure (B = 0.170, *P* = 0.666), disease duration (B = 0.009, *P* = 0.712), and FCI score (B = 0.010, *P* = 0.945)—were not independently significant in this sample, supporting their inclusion as confounders rather than direct predictors. Gender (B = 0.001, *P* = 0.997) and education level (all *P* > 0.10) were also non-significant in the adjusted model. The regression coefficients and 95% confidence intervals for all predictors in the multivariable model are visualized in Figure 2.

**Figure 2:**
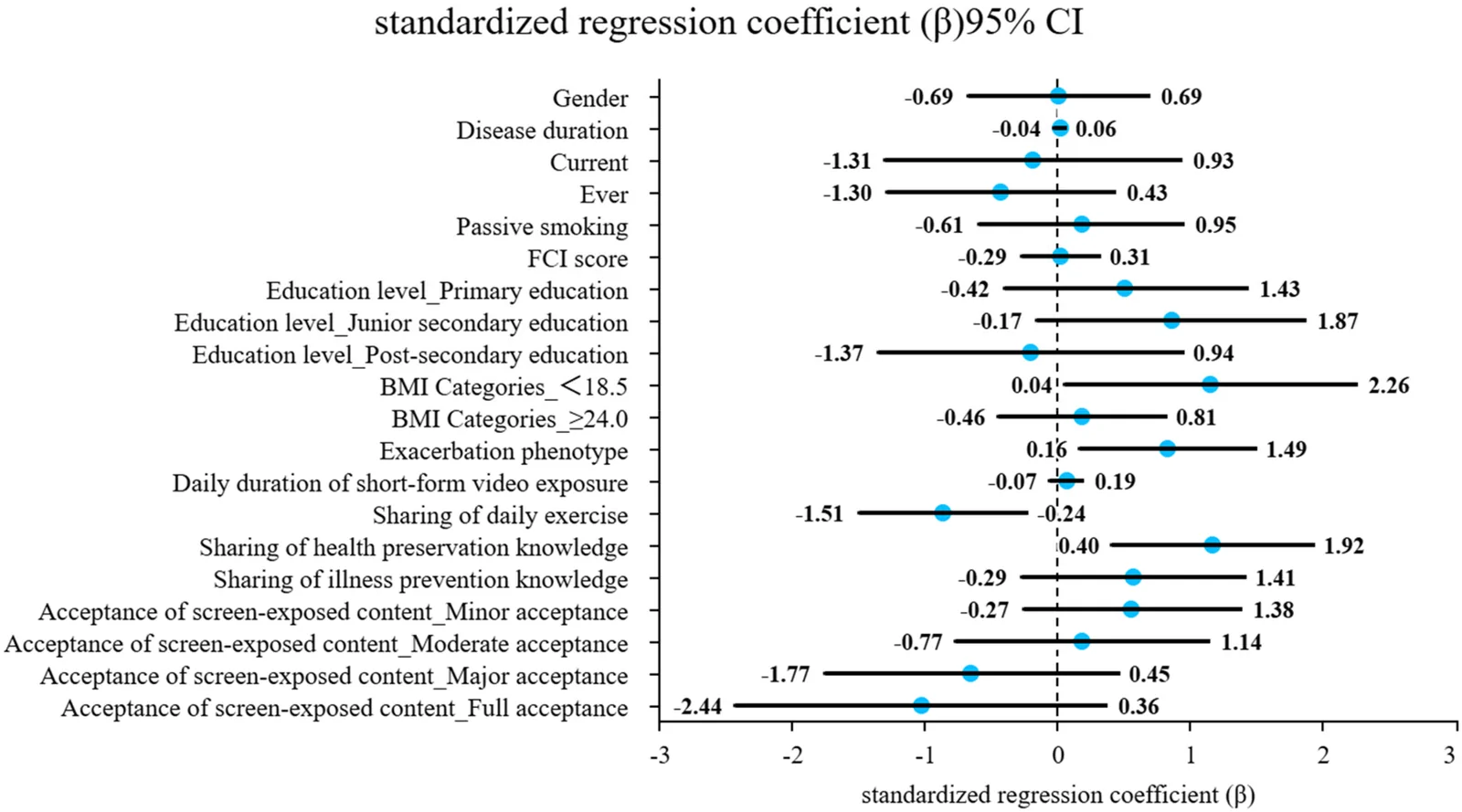
Forest plot of multivariable regression coefficients.

### 3.4 Hierarchical regression analysis

Table 5 presents the results of the hierarchical regression analysis with three strata. Stratum 1 (demographic and clinical covariates only) was statistically significant [F(12, 194) = 1.849, *P* = 0.043], explaining 10.3% of the variance in BCSS (R^2^ = 0.103, adjusted R^2^ = 0.047). Within this stratum, being underweight (B = 1.536, *P* = 0.006), junior secondary education (B = 1.391, *P* = 0.006), and exacerbation phenotype ≥2 (B = 0.706, *P* = 0.038) were significantly associated with higher BCSS scores.

**Table 5:** Results of hierarchical regression analysis (*n*=207)**.**

Variables	Stratum 1					Stratum 2					Stratum 3				
	*B*	S.E	*t*	*p*	*β*	*B*	S.E	*t*	*p*	*β*	*B*	S.E	*t*	*p*	*β*
Constant	6.898**	0.959	7.195	<0.001	-	6.385**	1.021	6.251	<0.001	-	6.489**	1.003	6.472	<0.001	-
Gender															
Female (Ref.)															
Male	−0.217	0.357	−0.608	0.544	−0.048	−0.185	0.356	−0.520	0.604	−0.041	0.001	0.350	0.003	0.997	<0.001
Disease duration	0.007	0.025	0.279	0.781	0.020	0.010	0.025	0.410	0.682	0.030	0.009	0.024	0.370	0.712	0.026
Smoking status															
Never (Ref.)															
Ever	−0.458	0.456	−1.005	0.316	−0.082	−0.438	0.455	−0.962	0.337	−0.078	−0.434	0.440	−0.987	0.325	−0.077
Current	−0.165	0.577	−0.285	0.776	−0.023	−0.211	0.577	−0.366	0.715	−0.030	−0.191	0.569	−0.335	0.738	−0.027
Passive smoking															
No (Ref.)															
Yes	−0.036	0.403	−0.090	0.929	−0.007	0.008	0.404	0.020	0.984	0.002	0.170	0.393	0.432	0.666	0.034
FCI score	0.085	0.146	0.585	0.559	0.050	0.157	0.154	1.017	0.310	0.091	0.010	0.151	0.069	0.945	0.006
Education level															
No formal (Ref.)															
Primary	0.873	0.469	1.860	0.064	0.154	0.792	0.471	1.680	0.095	0.140	0.508	0.470	1.081	0.281	0.090
Junior	1.391**	0.500	2.780	0.006	0.235	1.217*	0.514	2.370	0.019	0.206	0.847	0.517	1.639	0.103	0.143
Post-secondary	0.312	0.552	0.565	0.572	0.045	0.002	0.592	0.004	0.997	<0.001	−0.214	0.585	−0.366	0.715	−0.031
BMI															
18.5–23.9 (Ref.)															
＜18.5	1.536**	0.556	2.761	0.006	0.203	1.448*	0.558	2.594	0.010	0.191	1.150*	0.561	2.049	0.042	0.152
≥24.0	0.279	0.333	0.837	0.403	0.061	0.253	0.333	0.759	0.449	0.055	0.178	0.322	0.554	0.580	0.039
Exacerbation phenotype															
0–1 (Ref.)															
≥2	0.706*	0.339	2.084	0.038	0.151	0.751*	0.339	2.213	0.028	0.161	0.823*	0.337	2.447	0.015	0.176
Daily duration of short-form video exposure						0.091	0.064	1.428	0.155	0.130	0.059	0.066	0.886	0.377	0.084
Sharing of daily exercise															
No (Ref.)															
Yes											−0.876**	0.324	−2.704	0.007	−0.196
Sharing of health preservation															
No (Ref.)															
Yes											1.163**	0.386	3.009	0.003	0.227
Sharing of illness prevention															
No (Ref.)															
Yes											0.562	0.430	1.307	0.193	0.102
Acceptance of screen-exposed content															
Complete rejection (Ref.)															
Minor acceptance											0.556	0.419	1.325	0.187	0.112
Moderate acceptance											0.183	0.485	0.377	0.707	0.036
Major acceptance											−0.657	0.563	−1.168	0.244	−0.094
Full acceptance											−1.036	0.710	−1.460	0.146	−0.108
R-squared	0.103					0.112					0.220				
Adjust R-squared	0.047					0.052					0.136				
F Test	*F* (12,194)=1.849,*p*=0.043					*F* (13,193)=1.873,*p*=0.035					*F* (20,186)=2.627,*p*=<0.001				
ΔR-squared	0.103					0.009					0.108				
ΔF	*F* (12,194)=1.849,*p*=0.043					*F* (1,193)=2.040,*p*=0.155					*F* (7,186)=3.689,*p*=0.001				

Dependent Variable = BCSS; *p<0.05 **p<0.01.

Stratum 2 added daily short-form video viewing duration to the clinical covariate model. The incremental contribution of duration was small and non-significant [*Δ*R^2^ = 0.009; *Δ*F (1, 193) = 2.040; *P* = 0.155], confirming that viewing duration alone did not significantly improve the prediction of BCSS beyond established clinical factors.

Stratum 3 added the content type variables and acceptance of screen-exposed content to Stratum 2. The addition of these variables resulted in a statistically significant improvement in model fit [*Δ*R^2^ = 0.108; *Δ*F(7, 186) = 3.689; *P* = 0.001], indicating that short-form video content and acceptance characteristics explained an additional 10.8% of the variance in BCSS scores beyond clinical covariates and viewing duration. The final model (Stratum 3) explained 22.0% of the variance [R^2^ = 0.220, adjusted R^2^ = 0.136; F(20, 186) = 2.627; *P* < 0.001]. Within Stratum 3, health preservation content (B = 1.163, *β* = 0.227, *P* = 0.003), daily exercise content (B = −0.876, *β* = −0.196, *P* = 0.007), and exacerbation phenotype ≥2 (B = 0.823, *β* = 0.176, *P* = 0.015) were independently associated with BCSS. Illness prevention content (B = 0.562, *P* = 0.193) and daily viewing duration (B = 0.059, *P* = 0.377) remained non-significant, and the individual acceptance categories did not reach significance (all *P* > 0.12), although the overall pattern of coefficients was consistent with the non-linear relationship identified through RCS analysis.

### 3.5 Restricted cubic spline analysis

To further explore the non-linear relationships between continuous short-form video exposure variables and BCSS scores, restricted cubic spline regression with 4 knots was employed (Figures 3, 4).

**Figure 3:**
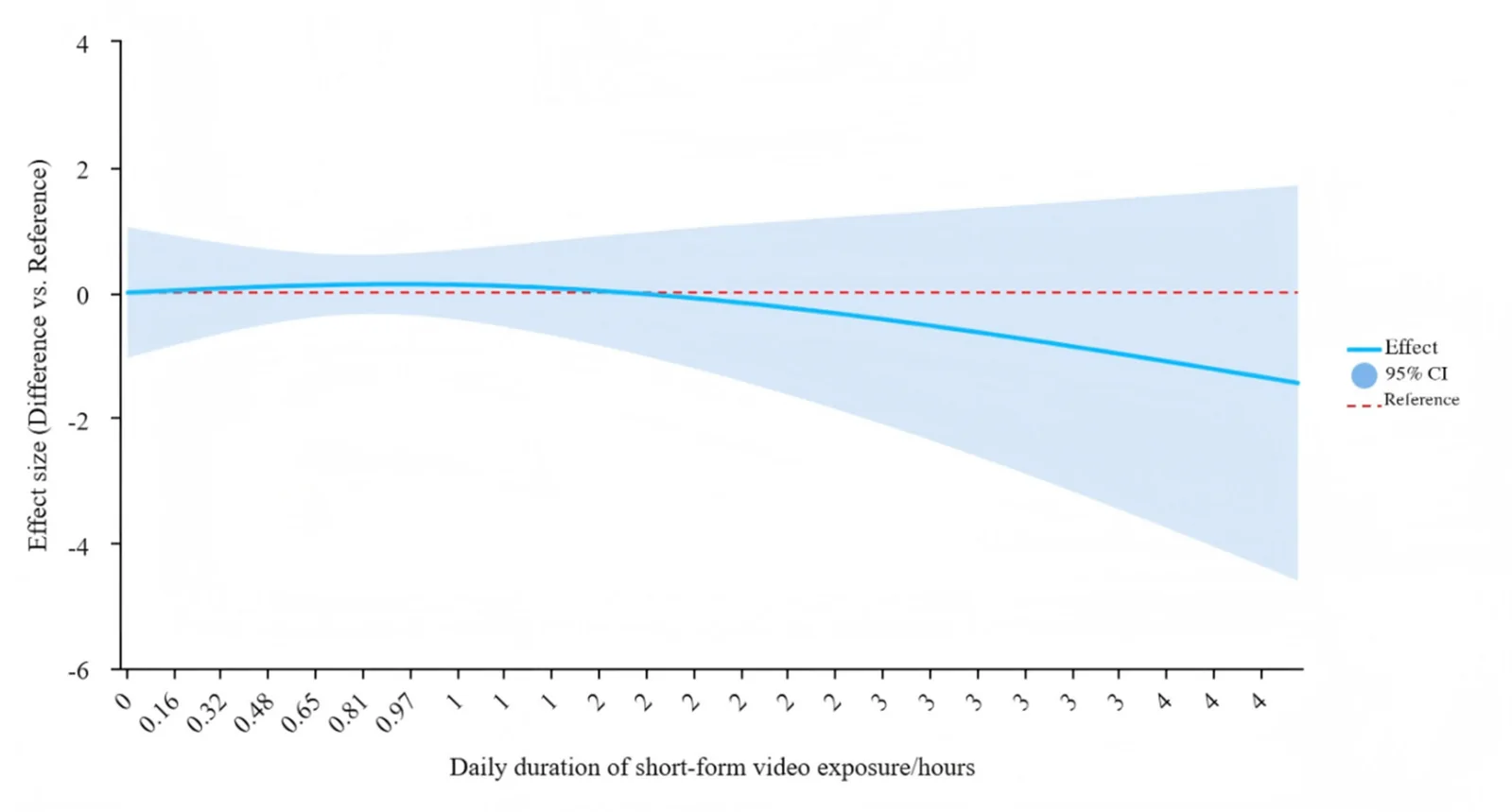
Restricted cubic spline curve for the association between daily short-form video exposure and effect estimates.

**Figure 4:**
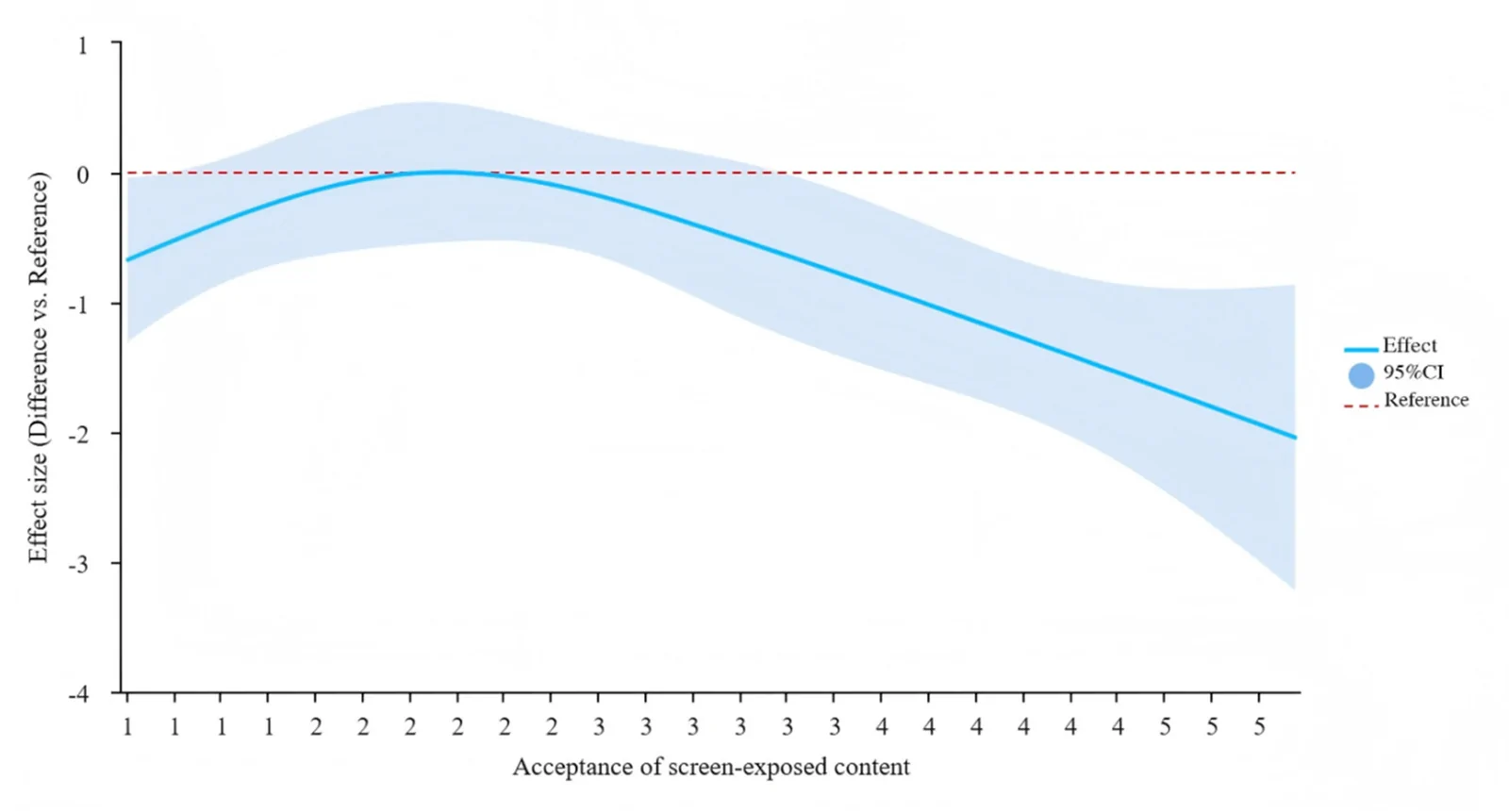
Restricted cubic spline curve for the association between acceptance of screen-exposed content and effect estimates.

For daily short-form video exposure duration (Figure 3), the RCS curve suggested a non-monotonic relationship with BCSS. The overall association between duration and BCSS was not statistically significant (P for overall association = 0.344), and the non-linearity test was also non-significant (P for non-linearity > 0.05), indicating that the duration-BCSS relationship did not deviate significantly from linearity after multivariable adjustment.

For acceptance of screen-exposed content (Figure 4), the RCS curve demonstrated a clear inverted U-shaped pattern. The spline curve (Figure 4) showed that the predicted BCSS increased as acceptance scores rose from low to low-moderate levels, reaching a peak at a fitted acceptance value of approximately 2.05 (on the 1–5 ordinal scale). This peak represents the point at which the spline-estimated BCSS was highest relative to the reference (complete rejection). Thereafter, as acceptance continued to increase, the predicted BCSS declined progressively, indicating a non-monotonic relationship. The estimated BCSS difference between the peak and the reference (acceptance = 1) was 1.02 (95% CI: −0.31 to 2.35), though this difference did not reach statistical significance in pairwise comparisons, likely due to limited power for subgroup contrasts. The overall non-linear pattern was statistically significant (P for non-linearity = 0.025), supporting a complex association that is not simply monotonic. The test for non-linearity was statistically significant (P for non-linearity = 0.025), supporting a non-linear pattern suggestive of an inverted U-shaped relationship, although this exploratory finding requires confirmation in future studies with more granular measurement of acceptance.

Sensitivity analyses treating acceptance as an ordered categorical variable (using orthogonal polynomial contrasts) were performed to assess the robustness of the non-linear pattern. The quadratic term was statistically significant (*P* = 0.013), consistent with the inverted U-shaped pattern identified by the RCS analysis. This sensitivity analysis supports the non-linear relationship between acceptance and BCSS, although the RCS approach provides a more flexible characterization of the curve's shape.

## 4 Discussion

### 4.1 Principal findings

This study is, to our knowledge, the first to examine the associations between multidimensional short-form video exposure characteristics and respiratory symptom severity in patients with COPD. Three principal findings emerged. First, the content type of short-form video exposure—rather than daily viewing duration—was significantly and independently associated with BCSS scores, explaining an additional 10.8% of symptom variance beyond clinical covariates and exposure duration. Second, exposure to health preservation content which, based on the existing literature on Chinese social media, often contains non-evidence-based claims was associated with significantly worse respiratory symptoms (B = 1.163, *P* = 0.003), while exposure to daily exercise content showed a protective inverse association (B = −0.876, *P* = 0.007) that emerged only after multivariable adjustment, consistent with a suppression effect (41). Third, the non-linear pattern observed for acceptance of screen-exposed content, with BCSS scores peaking at moderate acceptance (score ≈ 2.051) and declining thereafter (P for non-linearity = 0.025), suggests a complex association that is not simply monotonic. Together, these findings support a content-aware conceptualization of the health correlates of short-form video exposure and suggest that interventions targeting content evaluation skills—rather than simply reducing screen time—may be more effective for this population.

### 4.2 Health preservation content and respiratory symptoms

The positive association between exposure to health preservation content and higher BCSS scores (B = 1.163, *β* = 0.227, *P* = 0.003) represents the most clinically meaningful finding of this study. This association persisted after adjusting for a comprehensive set of clinical confounders including smoking status, secondhand smoke exposure, disease duration, comorbidity burden, exacerbation phenotype, and BMI, suggesting that the relationship is not attributable to differences in these established determinants of symptom severity. The most plausible interpretation is that health preservation content on Chinese short-form video platforms frequently contains non-evidence-based or potentially misleading health recommendations—such as unvalidated dietary therapies, meridian massage techniques, or traditional remedies that may delay or replace evidence-based pharmacological treatment (6, 7). Patients exposed to such content may alter their self-management behaviors in ways that are inconsistent with clinical guidelines (e.g., substituting prescribed medications with traditional remedies, delaying symptom escalation, or adopting inappropriate dietary restrictions), potentially contributing to poorer symptom control (38). This interpretation is consistent with prior work on health misinformation in social media, which has documented that exposure to non-evidence-based health content can influence patients' self-management decisions, potentially leading to delayed care-seeking, inappropriate treatment substitution, or adoption of unverified practices (6, 7, 38). From a theoretical perspective, the Elaboration Likelihood Model (ELM) (17, 39) predicts that individuals with lower eHealth literacy may be more susceptible to persuasive but non-evidence-based messages when processing health information through peripheral rather than central routes. The ELM is invoked here as an interpretive framework for understanding the observed association, not as a mechanism that was directly tested. However, we emphasize that the cross-sectional design precludes causal inference; reverse causality remains plausible—patients with more severe symptoms may be more likely to seek out health preservation content as a complementary strategy—and unmeasured confounding (e.g., health literacy, personality traits) may also contribute to the observed association.

It is important to note that the term “health preservation” (yangsheng) in the Chinese context encompasses a broad range of practices rooted in traditional Chinese medicine philosophy. While some yangsheng practices (e.g., tai chi, breathing exercises) may have evidence-based benefits, the term is also frequently co-opted by content creators disseminating unverified or commercially motivated health claims (40). The heterogeneity within this content category is a limitation of the current study, and future research should employ more granular content analysis to differentiate evidence-based from non-evidence-based yangsheng content.

### 4.3 Suppression effect of daily exercise content

The finding that daily exercise content was not significantly associated with BCSS in univariate analysis (*P* = 0.119) but became significantly and inversely associated after multivariable adjustment (B = −0.876, *P* = 0.007) is consistent with a classic suppression effect (37). In suppression, a variable that is not a significant bivariate predictor becomes a significant predictor in the multivariable model because it suppresses variance in another predictor that is irrelevant to the outcome, thereby enhancing the predictive validity of the model. In this context, the protective association of exercise content may have been masked in univariate analysis by confounding with other variables—for example, patients with more severe symptoms (higher BCSS) may be less likely to engage with exercise content (creating a positive confounding that masks the protective effect), or exercise content viewers may also tend to view health preservation content (creating a negative confounding). After adjustment for these correlated variables, the true protective association of exercise content was revealed. In more concrete terms, patients with worse respiratory symptoms were less likely to watch exercise-related content, which artificially diluted the apparent benefit of exercise content in the crude analysis; once this pattern was statistically adjusted for, the genuine protective effect of exercise content on respiratory symptoms became evident. This finding aligns with the extensive literature on the benefits of physical activity and pulmonary rehabilitation for COPD outcomes (41–43) and suggests that short-form video platforms may serve as a useful vehicle for delivering exercise-promoting content to patients with COPD, provided that such content is evidence-based and appropriately tailored.

### 4.4 Non-Linear acceptance–symptom relationship

The inverted U-shaped relationship between acceptance of screen-exposed content and BCSS scores (P for non-linearity = 0.025), with symptom severity peaking at moderate acceptance (score ≈ 2.051) and declining at higher acceptance levels, represents a novel finding that extends our third hypothesis. Several non-mutually-exclusive interpretations may account for this pattern. First, moderate acceptance may reflect a passive, uncritical consumption style in which viewers are sufficiently engaged to internalize content recommendations but lack the critical evaluation skills to distinguish evidence-based from non-evidence-based information, potentially leading to adoption of harmful self-management behaviors. However, the interpretation of this inverted U-shaped pattern warrants caution, as multiple competing explanations are plausible. One interpretation is that moderate acceptance reflects uncritical adoption of health recommendations\u2014including potentially misleading ones\u2014whereas full acceptance may reflect more active, discerning engagement in which viewers critically evaluate content before accepting it, resulting in preferential adoption of beneficial recommendations and rejection of harmful ones (19–22). This interpretation is consistent with the transactional model of eHealth literacy (20), which emphasizes that eHealth literacy is a dynamic, context-dependent process. Alternatively, the declining BCSS at higher acceptance levels may reflect a selection effect—patients with milder symptoms and better self-management capacity may be more accepting of health content—or reverse causality, whereby patients with better symptom control are more receptive to health information. A second alternative is that \u201cfull acceptance\u201d does not represent critical engagement at all, but rather passivity or compliance\u2014patients who uniformly accept all content may be less discriminating and less likely to act on any specific recommendation, resulting in no meaningful behavioral change and thus no impact on symptoms. A third possibility is that unmeasured confounders such as health literacy, digital literacy, and social support may underlie both acceptance patterns and symptom outcomes, such that the observed association reflects these upstream factors rather than a causal effect of content acceptance itself. The cross-sectional design precludes distinguishing between these mechanisms, and future research incorporating validated eHealth literacy instruments (e.g., the eHEALS or DHLI) (44, 45) and longitudinal designs would help clarify the directionality and moderators of this relationship.

### 4.5 Non-Significance of daily exposure duration

The finding that daily viewing duration was not independently associated with BCSS in the multivariable model (B = 0.059, *P* = 0.377) and did not significantly improve model fit when added to clinical covariates (*Δ*R^2^ = 0.009, *P* = 0.155) is consistent with our first hypothesis—that content type, rather than duration, would be the more salient correlate of respiratory symptom outcomes. This result challenges the predominant assumption in digital media health research that exposure duration is the primary driver of health effects (11, 12) and supports a more nuanced, content-aware conceptualization of the health correlates of short-form video consumption. The non-monotonic univariate pattern observed for exposure duration (with the highest BCSS scores at 6–7 h/day and lower scores at ≥8 h/day), while not statistically significant in adjusted analyses, may reflect unmeasured confounding: the small subgroup with the highest exposure duration (*n* = 12) may differ systematically from the remainder of the sample in ways not captured by the measured covariates. Furthermore, the measurement of exposure duration through self-report, which is subject to recall bias and social desirability effects (46), may have attenuated the true association. Future studies employing objective screen-time tracking or ecological momentary assessment methods would provide more precise exposure quantification. Notably, the univariate pattern of an inverted U-shape for daily viewing duration (with peak BCSS at 6–7 h/day) was attenuated after adjustment for content type and acceptance variables. This suggests that the apparent duration-related pattern observed in crude analyses may be largely attributable to confounding by content characteristics, rather than reflecting a direct dose-response effect of viewing time itself.

### 4.6 Comparison with existing literature

The present study contributes to a nascent but rapidly growing body of research on short-form video and health outcomes (11, 12, 18, 47). To our knowledge, this is the first study to examine the association between short-form video content type and respiratory symptom severity in patients with COPD, building on prior studies in other chronic disease populations that have reported associations between short-form video exposure and disease-related outcomes (13). The finding that exposure to non-evidence-based health content may be associated with worse physical health outcomes is consistent with theoretical models of health communication (39, 48) and aligns with recent evidence on the prevalence of low-quality health information on social media platforms (5–7, 40). The protective inverse association of exercise content extends prior work demonstrating the benefits of physical activity promotion through digital platforms (41, 42) and suggests that not all short-form video content is associated with negative outcomes—a critical nuance for the development of balanced clinical recommendations and public health messaging.

### 4.7 Clinical and public health implications

These findings have several implications for clinical practice and public health policy. First, the assessment of digital media behaviors—including both the quantity and content type of short-form video exposure—should be considered for integration into routine nursing-led COPD management. Nurses, as frontline healthcare providers with frequent and sustained patient contact, are uniquely positioned to conduct brief digital health behavior assessments and provide tailored media literacy education during routine clinical encounters (49). Second, the results support the development and evaluation of content-aware digital health literacy interventions specifically designed for older adults with COPD. Such interventions should aim to enhance patients' ability to critically evaluate the credibility and evidence base of health content encountered on short-form video platforms, while simultaneously promoting engagement with high-quality, evidence-based content (50, 51). Third, these findings underscore the importance of engaging healthcare professionals and professional organizations in the creation and dissemination of accurate, engaging, and accessible COPD-related content on short-form video platforms, thereby improving the overall quality of the health information ecosystem to which patients are exposed (52). Fourth, collaboration with platform developers to implement content moderation strategies, such as health information quality labels or algorithmic prioritization of content from verified health professional accounts, represents a structural approach to mitigating the potential harms of health misinformation at the systems level (53). We note that eHealth literacy and misinformation exposure were not directly measured in this study; the implications above are therefore hypothesis-generating and should be confirmed in future work that directly measures these constructs.

### 4.8 Limitations

Several limitations of this study should be acknowledged. First, the cross-sectional design precludes causal inference; the observed associations may reflect reverse causality (i.e., patients with more severe symptoms actively seeking specific content types), unmeasured confounding (e.g., health literacy, personality traits, social support), or bidirectional relationships that cannot be disentangled in the current design. Throughout this paper, we have used associational rather than causal language; however, even associational findings from cross-sectional data should be interpreted with caution regarding temporal ordering. In addition, acceptance of screen-exposed content was measured using a single-item ordinal scale, which limits its reliability and its ability to capture the multidimensional nature of acceptance; acceptance was also treated as a continuous variable in the spline analysis despite its ordinal nature, although sensitivity analyses treating it as categorical were consistent. Furthermore, several constructs central to the ELM frameworks (e.g., eHealth literacy, and information-processing pathways) and exposure to misinformation were not directly measured, and thus the theoretical mechanisms and the characterization of health preservation content as potentially non-evidence-based remain interpretive rather than empirically tested in this study.

Second, the study sample was recruited from hospital outpatient clinics in Yinchuan, a single city in northwestern China, using convenience sampling. This sampling strategy introduces potential selection bias: hospital-based participants may represent patients with more severe disease, greater healthcare-seeking motivation, or different digital media usage patterns compared with community-dwelling COPD patients who do not regularly attend clinics. Furthermore, the geographic restriction to one city limits the generalizability of findings to other regions with different cultural contexts, healthcare systems, or patterns of short-form video use. Results should not be extrapolated to the broader population of community-dwelling COPD patients without replication in more representative, multi-region samples.

Third, short-form video exposure was measured using a self-designed questionnaire that, despite acceptable psychometric properties (Cronbach *α* = 0.78, CVI = 0.85) and a content validation process involving expert consensus (≥50% agreement) and small-scale content verification (82% concordance with platform tags), has not been extensively validated against objective measures of media exposure. Self-reported screen time is known to correlate only modestly with device-logged measures. The binary content-type variables may not capture the nuanced patterns of content engagement (e.g., frequency, depth of engagement, active vs. passive consumption). Additionally, the single-item measurement of acceptance limits reliability compared with multi-item scales; future research should employ validated instruments such as the eHEALS or DHLI to capture the multidimensional construct of eHealth literacy.

Fourth, pulmonary function data (FEV₁% predicted) were not available for all participants and could not be included as a covariate. FEV₁% predicted is a key determinant of symptom severity in COPD (1), and its absence means that the regression models may not fully account for differences in airflow obstruction severity across participants. This limitation means that the observed associations between short-form video exposure variables and BCSS could potentially be confounded by unmeasured disease severity; however, the inclusion of exacerbation phenotype (a clinically relevant proxy for disease severity) as a covariate partially mitigates this concern. Future studies should include objective pulmonary function measures to control for this important confounder.

Fifth, the relatively modest overall model R^2^ of 0.220 indicates that a substantial proportion of variance in respiratory symptom severity remains unexplained by the variables included in this study. Important unmeasured factors such as current pharmacological treatment regimens, engagement with pulmonary rehabilitation, and detailed dietary and physical activity patterns likely contribute to symptom variance and should be included in future research.

Sixth, the algorithm-driven nature of content recommendation on short-form video platforms means that the content to which participants are exposed is not randomly assigned but is shaped by their prior viewing behavior, engagement patterns, and platform algorithms, introducing potential algorithmic confounding that is difficult to measure or control for in observational designs.

Seventh, the BCSS, while validated and widely used, captures only three respiratory symptom dimensions and does not assess other clinically relevant outcomes such as exercise capacity, health-related quality of life, or exacerbation frequency—all of which may be differentially associated with short-form video exposure.

Eighth, content categories were broad and may encompass heterogeneous sub-types; for example, “health preservation knowledge” could range from benign dietary advice to potentially harmful treatment recommendations. More granular content analysis methods, including natural language processing or manual coding of viewed content, would provide greater specificity.

Ninth, the achieved sample of 207 exceeds the formal a-priori requirement of 196 (based on G*Power for f^2^ = 0.15, *α*=0.05, power=0.80), providing adequate statistical power for detecting medium-to-large effects. Although this sample is marginally below the heuristic target of 210 (≈10 observations per predictor) derived from the 10–15 events-per-predictor rule, the observed overall effect (R^2^ = 0.22, f^2^ ≈ 0.28) is substantially larger than the assumed medium effect, further supporting adequate power for the primary analyses. We acknowledge that the effective ratio of ≈9.9 observations per predictor is slightly below the conventional 10:1 rule, which represents a minor limitation with respect to detecting very small effects; however, the *post-hoc* power for the observed effect size is well above 80%.

## 5 Conclusion

This cross-sectional study demonstrates that among patients with COPD, the content type of short-form video exposure—rather than exposure duration alone—is significantly associated with respiratory symptom severity. Health preservation content, which based on the existing literature on Chinese social media often contains non-evidence-based claims, was associated with worse symptoms, while daily exercise content showed a protective inverse association. The non-linear relationship between content acceptance and symptom severity was complex and not simply monotonic, suggesting that a single direction of association cannot be assumed; however, this pattern warrants confirmation in future studies with more granular measurement of acceptance and longitudinal designs.

## Data Availability

The raw data supporting the conclusions of this article will be made available by the authors, without undue reservation.
